# CT-based radiomic phenotypes of lung adenocarcinoma: a preliminary comparative analysis with targeted next-generation sequencing

**DOI:** 10.3389/fmed.2023.1191019

**Published:** 2023-08-17

**Authors:** Xiaowen Liu, Ting Xu, Shuxing Wang, Yaxi Chen, Changsi Jiang, Wuyan Xu, Jingshan Gong

**Affiliations:** ^1^The Second Clinical Medical College, Jinan University, Shenzhen, China; ^2^Department of Radiology, Shenzhen People's Hospital (The Second Clinical Medical College of Jinan University, The First Affiliated Hospital of Southern University of Science and Technology), Shenzhen, China; ^3^Guangzhou Red Cross Hospital, Jinan University, Guangdong, China

**Keywords:** radiomics, consensus clustering, targeted therapy, immunotherapy, lung adenocarcinoma

## Abstract

**Objectives:**

This study aimed to explore the relationship between computed tomography (CT)-based radiomic phenotypes and genomic profiles, including expression of programmed cell death-ligand 1 (PD-L1) and the 10 major genes, such as epidermal growth factor receptor (EGFR), tumor protein 53 (TP53), and Kirsten rat sarcoma viral oncogene (KRAS), in patients with lung adenocarcinoma (LUAD).

**Methods:**

In total, 288 consecutive patients with pathologically confirmed LUAD were enrolled in this retrospective study. Radiomic features were extracted from preoperative CT images, and targeted genomic data were profiled through next-generation sequencing. PD-L1 expression was assessed by immunohistochemistry staining (chi-square test or Fisher's exact test for categorical data and the Kruskal–Wallis test for continuous data). A total of 1,013 radiomic features were obtained from each patient's CT images. Consensus clustering was used to cluster patients on the basis of radiomic features.

**Results:**

The 288 patients were classified according to consensus clustering into four radiomic phenotypes: Cluster 1 (*n* = 11) involving mainly large solid masses with a maximum diameter of 5.1 ± 2.0 cm; Clusters 2 and 3 involving mainly part-solid and solid masses with maximum diameters of 2.1 ± 1.4 cm and 2.1 ± 0.9 cm, respectively; and Cluster 4 involving mostly small ground-glass opacity lesions with a maximum diameter of 1.0 ± 0.9 cm. Differences in maximum diameter, PD-L1 expression, and TP53, EGFR, BRAF, ROS1, and ERBB2 mutations among the four clusters were statistically significant. Regarding targeted therapy and immunotherapy, EGFR mutations were highest in Cluster 2 (73.1%); PD-L1 expression was highest in Cluster 1 (45.5%).

**Conclusion:**

Our findings provide evidence that CT-based radiomic phenotypes could non-invasively identify LUADs with different molecular characteristics, showing the potential to provide personalized treatment decision-making support for LUAD patients.

## 1. Introduction

Lung cancer is the most commonly malignant cancer worldwide and the main cause of cancer-related death (1, 2). Non-small cell lung cancer (NSCLC) is the main type of lung cancer, accounting for ~80–90% of all lung cancers, and lung adenocarcinoma (LUAD) has been identified as the primary histologic subtype (3). When LUAD progresses to an inoperable tumor in advanced stages, systemic chemotherapy is the only option. Unfortunately, response rates for platinum-based chemotherapy ranged only between 20% and 40% (4). Targeted therapy for some molecular abnormalities and immunotherapy eliciting T-cell immunoreactivity dramatically improve the survival of some LUAD patients and alter management regimens (5, 6). However, only a small proportion of patients with special molecular characteristics or tumor-immune microenvironments (TIMEs) respond to these therapies (7, 8). Therefore, knowledge of these metrics is needed for selecting patients who would benefit from targeted therapy or immunotherapy. Nevertheless, all of these metrics require an invasive approach to obtain tissue specimens through expensive, time-consuming, and labor-intensive laboratory and clinical testing, and because tumor molecular profiles or the TIME can evolve during treatment, this process may be repeated. In some clinical scenarios, obtaining tissue specimens is difficult. On the other hand, there are sampling errors for tissue-based biomarkers due to the heterogeneity of LUAD, especially the specimens obtained by biopsy (9). Therefore, it is necessary to find non-invasive surrogate biomarkers.

Radiomics, which extracts a large number of quantitative features from medical imaging with high throughput to translate digital images into a wealth of mineable data, is a promising discipline that bridges imaging and precision medicine (10, 11). Previous studies have shown that computed tomography (CT)-based radiomics can decode the molecular or immune characteristics of LUADs (12–14). To the best of our knowledge, only one study has probed the relationship between radiomics and genomic profiles of LUADs (15). The purpose of this study was to develop CT-based radiomic phenotypes using consensus clustering to predict the molecular characteristics and TIME of LUADs to facilitate patient selection for targeted therapies and immunotherapies.

## 2. Materials and methods

### 2.1. Patient population

**Figure 1:**
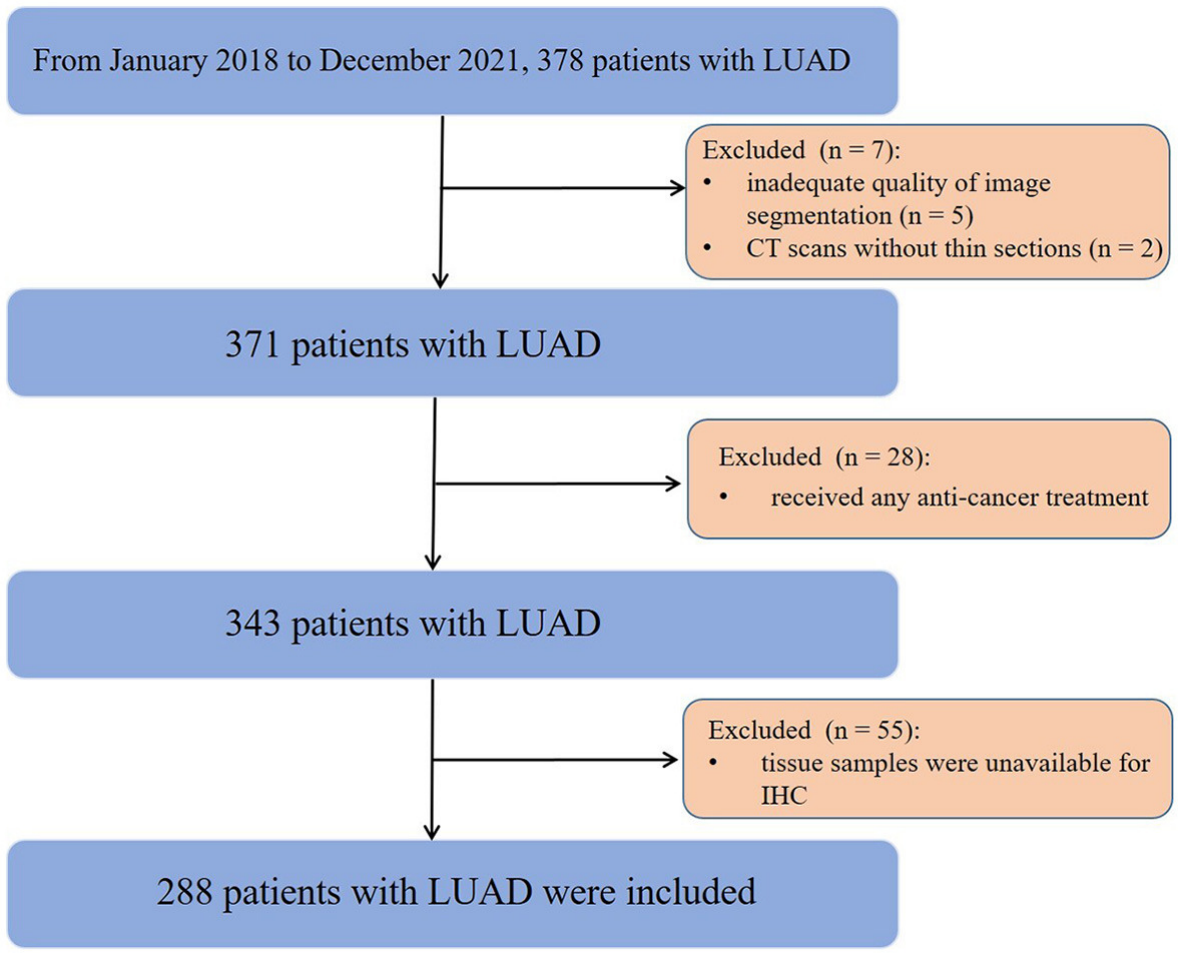
Flowchart of the patient selection process.

This study was approved by the Institutional Review Board, which waived the informed consent requirement due to its retrospective nature. From January 2018 to December 2021, a total of 378 consecutive patients with surgically pathologically confirmed LUAD were enrolled. The inclusion criteria were as follows: (1) successful retrieval of CT images from the picture archiving and communication system (PACS); (2) CT examinations performed within 3 months before surgery; and (3) diagnosis of LUAD with complete surgical resection (R0) and preoperative next-generation sequencing (NGS) data. The exclusion criteria included the following: (1) inadequate quality of image segmentation (*n* = 5); (2) CT scans without thin slices (*n* = 2); (3) received any anti-cancer treatment (*n* = 28); and (4) tissue samples were unavailable for immunohistochemistry (IHC) (*n* = 55). Finally, 288 patients (123 males, 165 females; median age, 58 years; interquartile range [IQR], 48–67 years) were eligible for this study (Figure 1). Demographic and clinical data included age, sex, family history, smoking status, carcinoembryonic antigen (CEA), carbohydrate antigen 125 (CA125), carbohydrate antigen 125 (CA199), clinical stage, and tumor mutational burden (TMB).

### 2.2. Immunohistochemistry

Formalin-fixed paraffin-embedded (FFPE) samples from LUADs were sliced at a thickness of 3–4 μm, and IHC was used to detect the expression of programmed cell death ligand 1 (PD-L1) in the FFPE samples. The PD-L1 test kit used 22C3 pharmDx (Dako Company). When using this antibody, only staining of the tumor cell membrane was considered, whereas positive staining of the cytoplasm was ignored. Some or all of the tumor cells expressing any linear or granular staining on the cell membrane were counted as positive. The tumor proportion score (TPS) is defined as the percentage of tumor cells stained with the PD-L1 membrane at any intensity. PD-L1 expression was dichotomized according to the TPS level. The widespread consensus is that TPS <1% is negative for expression but that TPS ≥1% is positive for expression, with the latter being appropriate for treatment with PD-L1 antibodies (16).

### 2.3. Targeted NGS and data processing

NGS was performed as previously described (17, 18). Tumor DNA and corresponding patient-matched blood DNA were extracted. TMB was defined as the total number of non-synonymous single-nucleotide or insertion/deletion mutations divided by the length in Mb of the coding region sequenced by each panel (0.98, 1.06, and 1.22 Mb in the 341-, 410-, and 468-gene panels, respectively) (19). The fraction of the genome altered (FGA) was defined as the fraction of log_2_ copy number variation (gain or loss) >0.2 divided by the size of the genome for which the copy number was profiled (20). A total of 520 genes closely related to cancer mechanisms and targeted therapies were detected, covering the full exonic regions of 310 genes and 210 hotspot mutation regions (exons, introns, or promoter regions) of 310 genes.

### 2.4. Non-contrast CT image acquisition

CT examinations were performed using a 128-detector CT scanner (Philips Brilliance iCT, Philips Medical Systems, Best, the Netherlands). The CT parameters were as follows: collimation of 0.625 mm × 128; tube voltage, 120 kV; and tube current, automatically adjusted. All CT images were reconstructed with a slice thickness of 1.0 mm and a gap of 0.5 mm using a lung kernel.

### 2.5. Assessment of non-contrast CT morphological features

Two radiologists with 5 and 12 years of experience in thoracic radiology reviewed the CT images and estimated the types of nodules [solid, part-solid, and ground-glass nodules (GGOs)] in consensus on our PACS. They were all blinded to the identity and clinical data of each subject. A consensus was reached prior to the assessment of CT morphological features. A total of 15 CT imaging features were evaluated, including CT location, tumor size, type of nodules, necrosis, vacuole sign, cavity sign, thickened pleura, pleural traction sign, pleural effusion, lymph node enlargement, vascular cluster sign, lobulation, spiculation, calcification, and air bronchograms.

The CT location was divided into the left upper lobe, lower lobe, right upper lobe, middle lobe, and lower lobe. Nodule types were categorized as GGO (GGO = 100%), part-solid (0% < GGO <100%), and solid (GGO = 0%) according to the proportion of ground glass. GGO was defined as a hazy increase in the lung window setting with the preservation of bronchial and vascular markings (21). The tumor size was assessed by the maximum diameter of the nodules. The vacuole sign was measured by a tumor diameter <5 mm with a hypointense radiolucent shadow, and the cavity sign was defined as a thick-walled cavity with a cavity wall larger than 3 mm. The two features of pleural invasion were pleural thickening and traction. Enlarged lymph nodes were defined as lymph nodes in the mediastinum with a short axis >10 mm. Lobulation was defined as the shallow wavy contour of a tumor's surface, with the exception of the portion adjacent to the pleura (22). Spiculation was defined as sharp linear projections in the targeted tumor lesion. Calcification on CT images was defined as the presence of high-density material in the tumor. Air bronchogram signs on CT images were defined as small foci or branches of air attenuation within the solid part of the tumor (23).

### 2.6. Radiomic feature extraction and consensus clustering

Digital Imaging and Communications in Medicine (DICOM) images were downloaded from PACS and transferred to a personal computer (PC) installed with ITK-SNAP version 3.6.0-beta (http://www.itksnap.org/). The two radiologists were blinded to all clinical and gene information and used ITK-SNAP to manually delineate LUAD lesions slice by slice, obtaining regions of interest (ROI) of the whole tumor in lung window settings. Intraclass correlation coefficients (ICCs) were used to exclude features with low reliability (ICCs < 0.75), and averages of the included feature values of the radiologists' segmentation were used for further analysis. The open-source software reconstructs the three-dimensional volumes of interest (VOIs) of whole tumors automatically. Radiomic features were extracted from the VOIs using the software Pyradiomics 3.0 Python package, which has recently been shown to align with the image biomarker Standardization Initiative. A total of 1,013 quantitative features were obtained from each patient's CT scan, consisting of 18 first-order statistical features, 14 shape features, 24 gray-level co-occurrence matrix (GLCM) features, 16 gray-level run-length matrix (GLRLM) features, 16 gray-level size zone matrix (GLSZM) features, 14 gray-level dependence matrix (GLDM) features, and 5 neighborhood gray-tone difference matrix (NGTDM) features. In addition, several image filters were applied to the original image to obtain corresponding derived images, including Wavelet, Square, Squareroot, and Laplacian of Gaussian (LoG).

**Figure 2:**
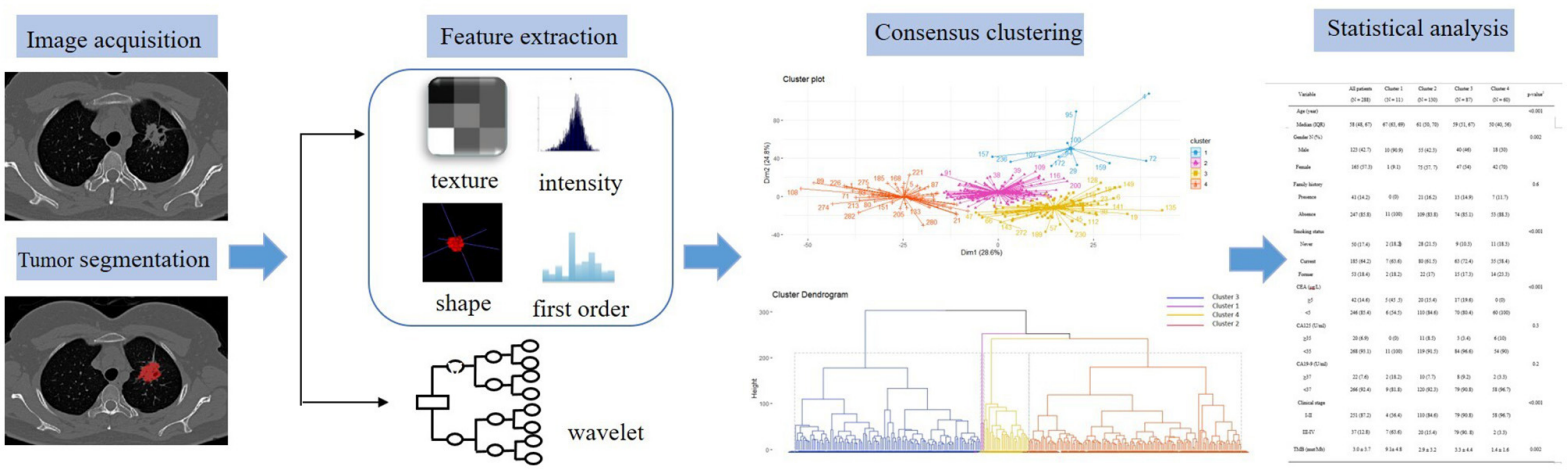
Flowchart showing the radiomic image analysis process.

**Figure 3:**
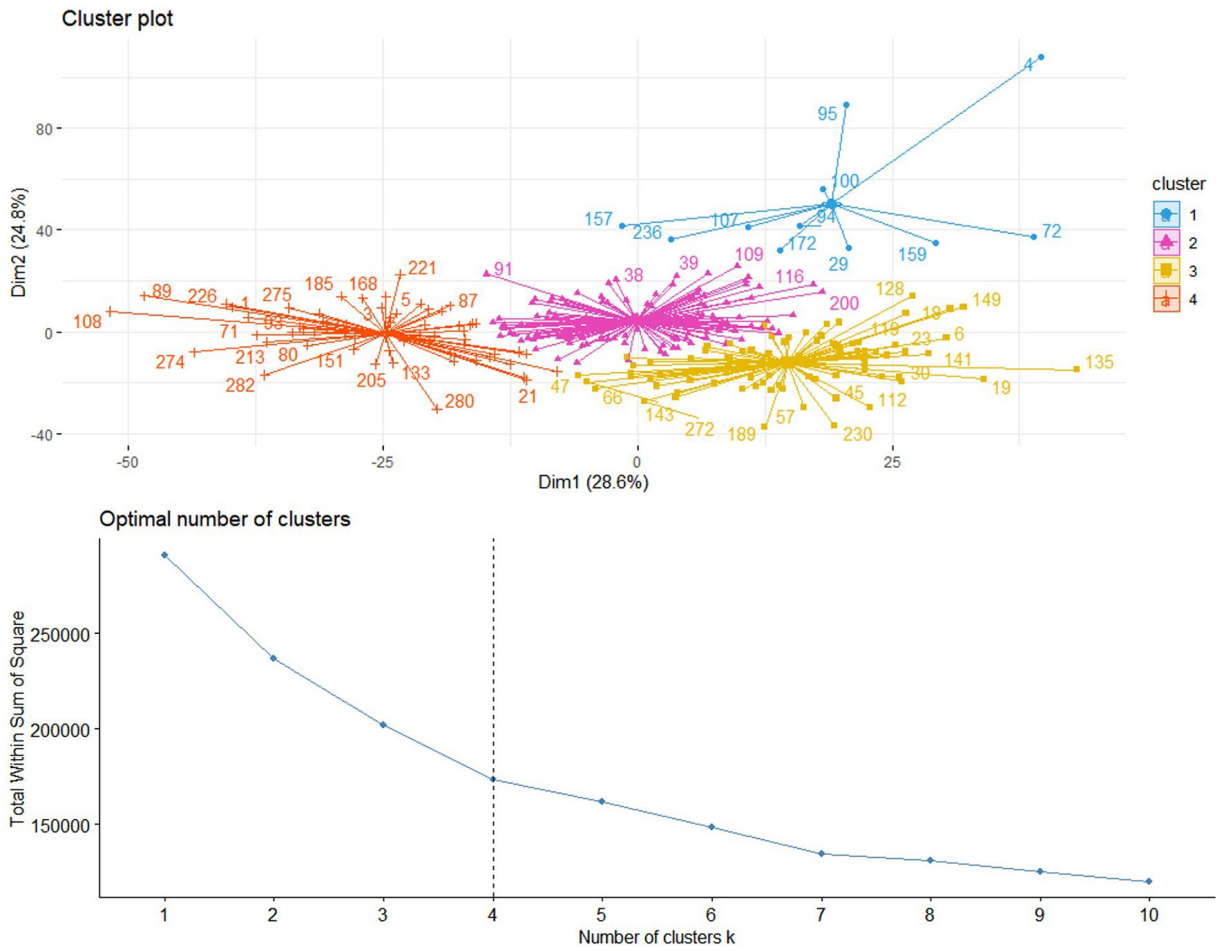
Based on the area change under the conditional density function curve, we observed that clustering separation was optimal at a *k*-value of 4. This value corresponded to a sharp decrease in the area change under the receiver operating characteristic curve, which suggested that after this *k*-value, further improvements in separability were negligible.

In this study, we utilized a consensus clustering approach to discover intrinsic radiomic subtypes of LUAD (Figure 2). Consensus clustering was performed using the “ConsensusClusterPlus” R package, which accomplished unsupervised clustering analysis to identify LUAD subgroups from 1,013 radiomic features without human intervention. During the clustering process, 80% of the samples were sampled 1,000 times by adopting the resampling iterations. The distance correlation between samples was calculated using the Euclidean distance, with the clustering algorithm using k-means for reliable subgroup classification (Figure 3). The optimal *k*-value, which corresponded to the most well-separated and stable cluster, was determined by a sharp decrease in the area change under the receiver operating characteristic curve. Further improvements in separability beyond this *k*-value were deemed insignificant. LUADs were effectively grouped into appropriate subgroups based on this k-value.

### 2.7. Statistical analyses

Statistical analysis was performed in R version 4.2.2 (R Foundation for Statistical Computing) and SPSS version 23.0. A *P*-value of <0.05 indicated statistical significance. We varied the number of clusters from 2 to 8 and selected the optimal number of clusters in the training cohort for unsupervised clustering. Clinical metrics, imaging characteristics, and genomic profiles of the final clusters were compared using the chi-square test or Fisher's exact test for categorical data and the Kruskal–Wallis test for continuous data. Continuous data are expressed as the mean ± SD or median (lower and upper quartiles), and categorical data are expressed as frequencies and percentages.

## 3. Results

### 3.1. Patient characteristics

Patient characteristics are listed in Table 1. Of the 288 eligible patients, the median age was 58 years (IQR, 48–67 years), 123 (42.7%) were men, and 165 (57.3%) were women. Most patients did not have a positive family history of lung cancer. The majority of patients were current (*n* = 185 [64.2%]) smokers. The mean TMB value was 3.0 ± 3.7 mutations per megabase (range: 0–31.9). In addition, most of the patients (87.2%) were in the early clinical stage (I–II). Differences in age, sex, and smoking status between the four clusters were statistically significant, and the CEA, clinical stage, and TMB among the four clusters were also statistically significant (*P* < 0.05).

**Table 1:** Patient characteristics.

**Variable**	**All patients (*N =* 288)**	**Cluster 1 (*N =* 11)**	**Cluster 2 (*N =* 130)**	**Cluster 3 (*N =* 87)**	**Cluster 4(*N =* 60)**	***p*-value^2^**
**Age (years)**						<0.001
Median (IQR)	58 (48, 67)	67 (63, 69)	61 (50, 70)	59 (51, 67)	50 (40, 56)	
**Gender** ***N*** **(%)**						0.002
Male	123 (42.7)	10 (90.9)	55 (42.3)	40 (46)	18 (30)	
Female	165 (57.3)	1 (9.1)	75 (57.7)	47 (54)	42 (70)	
**Family history**						0.6
Presence	41 (14.2)	0 (0)	21 (16.2)	13 (14.9)	7 (11.7)	
Absence	247 (85.8)	11 (100)	109 (83.8)	74 (85.1)	53 (88.3)	
**Smoking status**						<0.001
Never	50 (17.4)	2 (18.2)	28 (21.5)	9 (10.3)	11 (18.3)	
Current	185 (64.2)	7 (63.6)	80 (61.5)	63 (72.4)	35 (58.4)	
Former	53 (18.4)	2 (18.2)	22 (17)	15 (17.3)	14 (23.3)	
**CEA (**μ**g/L)**						<0.001
≥5	42 (14.6)	5 (45.5)	20 (15.4)	17 (19.6)	0 (0)	
<5	246 (85.4)	6 (54.5)	110 (84.6)	70 (80.4)	60 (100)	
**CA125 (U/ml)**						0.3
≥35	20 (6.9)	0 (0)	11 (8.5)	3 (3.4)	6 (10)	
<35	268 (93.1)	11 (100)	119 (91.5)	84 (96.6)	54 (90)	
**CA19-9 (U/ml)**						0.2
≥37	22 (7.6)	2 (18.2)	10 (7.7)	8 (9.2)	2 (3.3)	
<37	266 (92.4)	9 (81.8)	120 (92.3)	79 (90.8)	58 (96.7)	
**Clinical stage**						<0.001
I–II	251 (87.2)	4 (36.4)	110 (84.6)	79 (90.8)	58 (96.7)	
III–IV	37 (12.8)	7 (63.6)	20 (15.4)	8 (9.2)	2 (3.3)	
TMB (mut/Mb)	3.0 ± 3.7	9.1± 4.8	2.9 ± 3.2	3.3 ± 4.4	1.4 ± 1.6	0.002

IQR, interquartile range; CEA, carcinoembryonic antigen; CA125, carbohydrate antigen 125; CA19-9, carbohydrate antigen 19-9; TMB, tumor mutational burden.

### 3.2. CT findings of the LUADs

**Figure 4:**
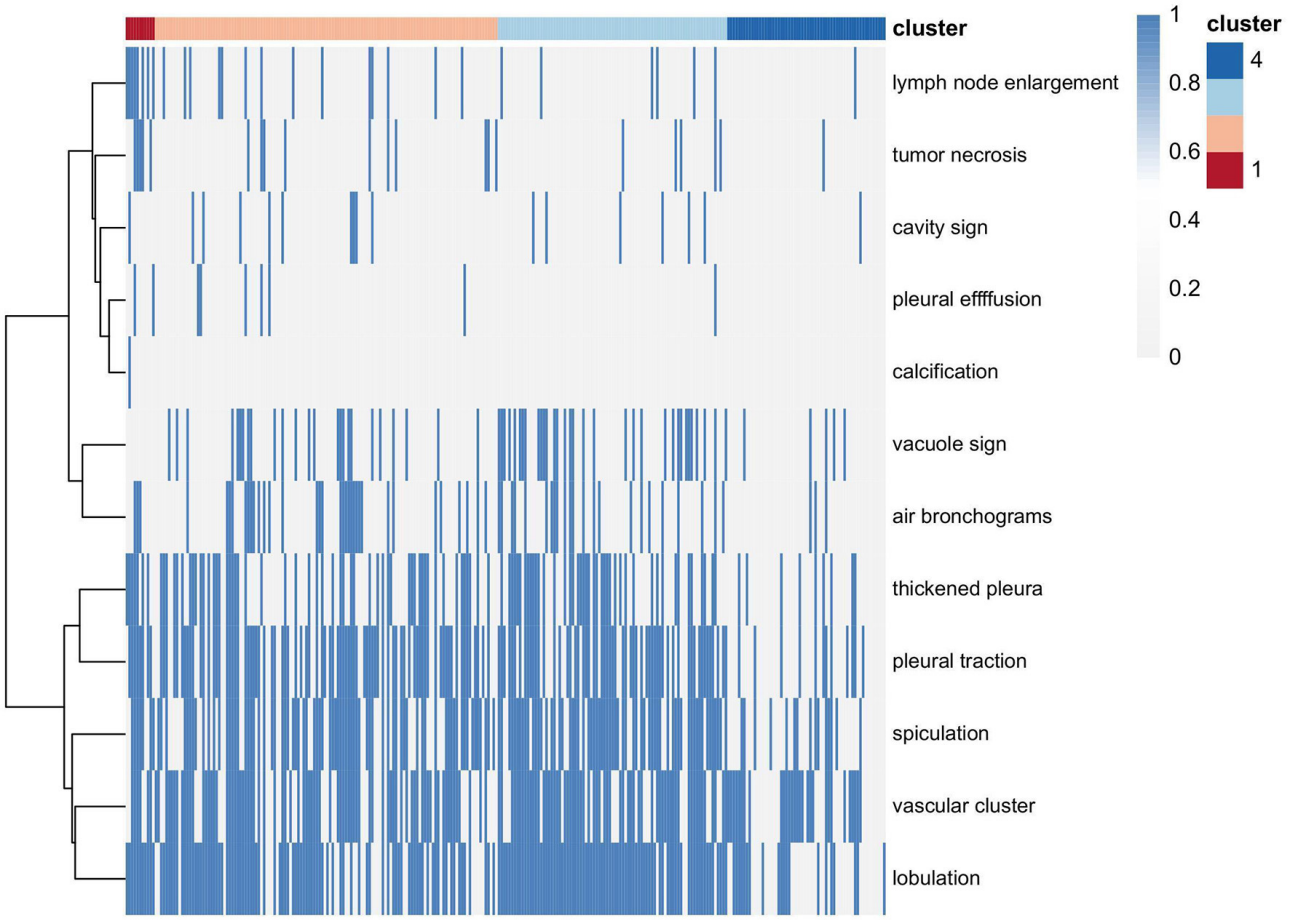
Distribution of CT morphological features in the four clusters.

The CT findings of LUADs are listed in Table 2. There were no statistically significant differences in CT location, cavity sign, pleural effusion, or calcification characteristics among the four clusters (*P* > 0.05) (Figure 4).

**Table 2:** CT morphological features of patients in the clusters.

**Variable**	**Overall (*N* = 288)**	**Cluster 1 (*N* = 11)**	**Cluster 2 (*N* = 130)**	**Cluster 3 (*N =* 87)**	**Cluster 4 (*N =* 60)**	***p*-value^2^**
**CT location**						0.856
Left upper lobe	85 (29.5)	5 (45.5)	42 (32.3)	24 (27.6)	15 (25)	
Left lower lobe	50 (17.3)	2 (18.2)	21 (16.2)	14 (16.1)	12 (20)	
Right upper lobe	77 (26.7)	4 (36.3)	31 (23.8)	24 (27.6)	18 (30)	
Right middle lobe	29 (10.1)	0 (0)	13 (10)	9 (10.3)	7 (11.7)	
Right lower lobe	47 (16.4)	0 (0)	23 (17.7)	16 (18.4)	8 (13.3)	
Maximum diameter (cm)^*^	2.3 ± 1.4	5.1 ± 2.0	2.1 ± 1.4	2.1 ± 0.9	1.0 ± 0.9	<0.001
**Type of nodules**						<0.001
Ground glass	102 (35.4)	0 (0)	42 (32.4)	15 (17.2)	45 (75)	
Part-solid	86 (29.9)	1 (9.1)	44 (33.8)	30 (34.5)	11 (18.3)	
Solid	100 (34.7)	10 (90.9)	44 (33.8)	42 (48.3)	4 (6.7)	
Tumor necrosis (–)	268 (92.7)	6 (55.5)	120 (92.3)	82 (94.2)	59 (98.3)	<0.001
Tumor necrosis (+)	21 (7.3)	5 (45.5)	10 (7.7)	5 (5.8)	1 (1.7)	
Vacuole sign (–)	225 (78.2)	11 (100)	105 (80.8)	54 (62)	55 (91.6)	<0.001
Vacuole sign (+)	63 (21.8)	0 (0)	25 (19.2)	33 (38)	5 (8.4)	
Cavity sign (–)	271 (94)	10 (90.9)	121 (93.1)	81 (93.1)	59 (98.3)	0.468
Cavity sign (+)	17 (6)	1 (9.1)	9 (6.9)	6 (6.9)	1 (1.7)	
Thickened pleura (–)	176 (61.2)	4 (36.4)	77 (59.2)	44 (50.5)	51 (85)	<0.001
Thickened pleura (+)	112 (38.8)	7 (63.6)	53 (40.8)	43 (49.5)	9 (15)	
Pleural traction sign (–)	125 (43.5)	3 (27.3)	46 (35.4)	28 (32.1)	48 (80)	<0.001
Pleural traction sign (+)	163 (56.5)	8 (72.7)	84 (64.6)	59 (67.9)	12 (20)	
Pleural effusion (–)	279 (96.9)	9 (81.8)	124 (95.4)	86 (98.9)	60 (100)	0.514
Pleural effusion (+)	9 (3.1)	2 (18.2)	6 (4.6)	1 (1.1)	0 (0)	
Lymph node enlargement (–)	259 (89.9)	3 (27.3)	116 (89.2)	81 (93.1)	59 (98.3)	<0.001
Lymph node enlargement (+)	29 (10.1)	8 (72.7)	14 (10.8)	6 (6.9)	1 (1.7)	
Vascular cluster sign (–)	109 (37.8)	5 (45.5)	49 (37.7)	24 (27.6)	31 (51.6)	0.028
Vascular Cluster Sign (+)	179 (62.2)	6 (54.5)	81 (62.3)	63 (72.4)	29 (48.4)	
Lobulation (–)	86 (29.9)	0 (0)	41 (31.5)	5 (5.8)	40 (66.6)	<0.001
Lobulation (+)	202 (70.1)	11 (100)	89 (68.5)	82 (94.2)	20 (33.4)	
Spiculation (–)	123 (42.8)	4 (36.4)	52 (40)	22 (25.3)	45 (75)	<0.001
Spiculation (+)	165 (57.2)	7 (63.6)	78 (60)	65 (74.7)	15 (25)	
Calcification (–)	287 (99.7)	11 (100)	130 (100)	86 (98.9)	60 (100)	0.5
Calcification (+)	1 (0.3)	0 (0)	0 (0)	1 (1.1)	0 (0)	
Air bronchograms (–)	227 (78.9)	8 (72.7)	98 (75.4)	64 (73.5)	57 (95)	0.002
Air bronchograms (+)	61 (21.1)	3 (27.3)	32 (24.6)	23 (26.5)	3 (5)	

### 3.3. Pathologic characteristics and gene profiles

**Figure 5:**
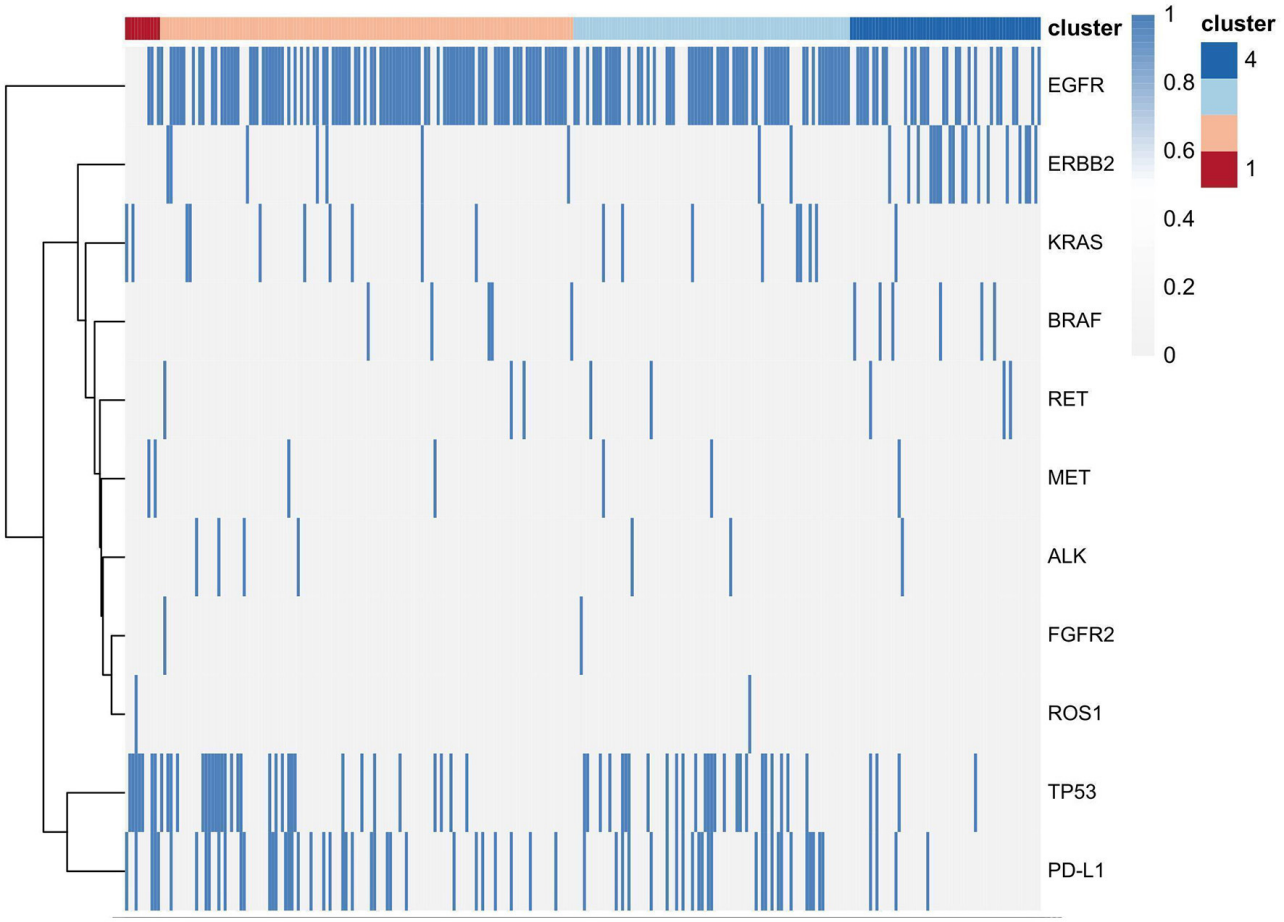
Several gene expression profiles in the four clusters.

Pathologic characteristics and gene profiles are listed in Table 3. There were significant differences in pathologic nodal status, pleural invasion, and vascular invasion (*P* < 0.05), but no significant difference was found in nerve invasion (*P* = 0.2). PD-L1 expression outcome was available for 288 patients, of whom 67 (23.2%) were positive for expression (TPS ≥ 1%). Epidermal growth factor receptor (EGFR) (*n* = 184 [63.9%]) and tumor protein 53 (TP53) (*n* = 66 [22.9%]) mutations were the most frequent actionable genomic alterations in our cohort (Figure 5).

**Table 3:** Tumor pathologic characteristics and gene expression.

**Variable**	**All patients (*N =* 288)**	**Cluster 1 (*N =* 11)**	**Cluster 2 (*N =* 130)**	**Cluster 3 (*N =* 87)**	**Cluster 4 (*N =* 60)**	***p*-value^2^**
**Pathologic nodal status**						0.002
Positive	27 (9.4)	4 (36.4)	12 (9.2)	9 (10.3)	2 (3.3)	
Negative	261 (90.6)	7 (63.6)	118 (90.8)	78 (89.7)	58 (96.7)	
**Visceral pleural invasion**						0.041
Positive	37 (12.8)	2 (18.2)	18 (13.8)	15 (17.2)	2 (3.3)	
Negative	251 (87.2)	9 (81.8)	112 (86.2)	72 (82.8)	58 (96.7)	
**Lymphovascular invasion**						< 0.001
Positive	33 (11.5)	4 (36.4)	16 (12.3)	13 (14.9)	0 (0)	
Negative	255 (88.5)	7 (66.6)	114 (87.7)	74 (85.1)	60 (100)	
**Pathological nerve invasion**						0.2
Positive	5 (1.7)	1 (9.1)	3 (2.3)	1 (1.1)	0 (0)	
Negative	283 (98.3)	10 (90.9)	127 (97.7)	86 (98.9)	60 (100)	
**PD-L1 expression**						0.003
TPS ≥ 1%	67 (23.2)	5(45.5)	33(25.4)	25(28.7)	4(6.67)	
**Actionable mutations**						
EGFR	184 (63.9)	3 (27.3)	95 (73.1)	59 (67.8)	27 (45)	< 0.001
TP53	66 (22.9)	7 (63.6)	29 (22.3)	26 (29.9)	4 (6.7)	< 0.001
ERBB2	27 (9.4)	0 (0)	7 (5.4)	2 (2.3)	18 (30)	< 0.001
KRAS	19 (6.6)	2 (18.2)	8 (6.1)	8 (9.2)	1 (1.7)	0.10
BRAF	11 (3.8)	0 (0)	5 (3.8)	0 (0)	6 (10)	0.017
RET	8 (2.8)	0 (0)	3 (2.3)	2 (2.3)	3 (5.0)	0.7
ALK	7 (2.4)	0 (0)	4 (3.1)	2 (2.3)	1 (1.7)	0.88
MET	7 (2.4)	2 (18.2)	2 (1.5)	2 (2.3)	1 (1.7)	0.052
FGFR2	2 (0.7)	0 (0)	1 (0.7)	1 (1.1)	0 (0)	0.857
ROS1	2 (0.7)	1 (9.1)	0 (0)	1 (1.1)	0 (0)	0.040

TPS, tumor proportion score; PD-L1, programmed cell death-ligand 1.

### 3.4. Consensus clustering associations

**Figure 6:**
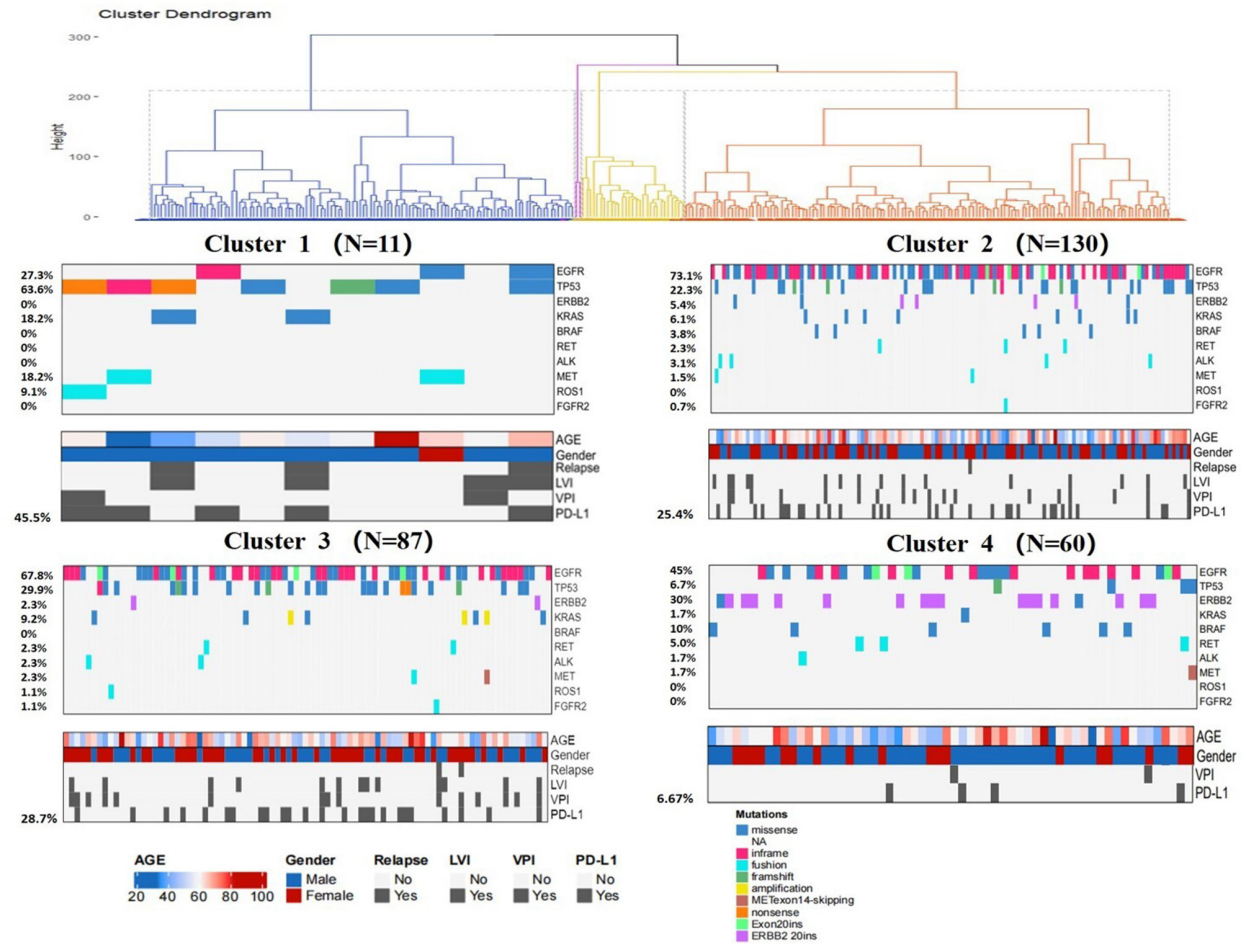
Clinical pathologic and genomic data for all LUADs. Cluster characteristics were compared using the chi-square test or Fisher's exact test for categorical data and the Kruskal–Wallis test for continuous data. LVI = lymphovascular invasion, VPI = visceral pleural invasion.

**Figure 7:**
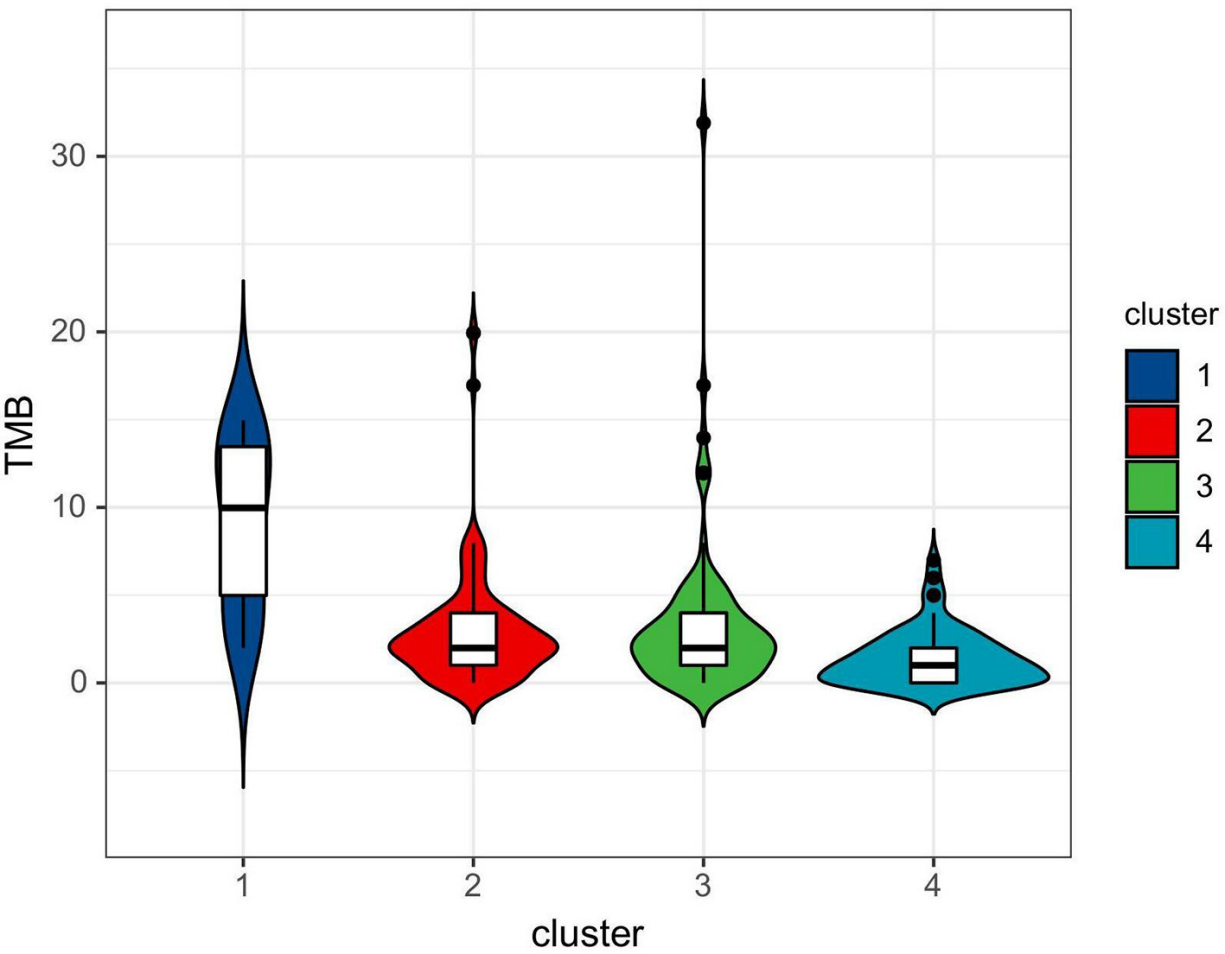
Violin plot of TMB among four clusters.

**Figure 8:**
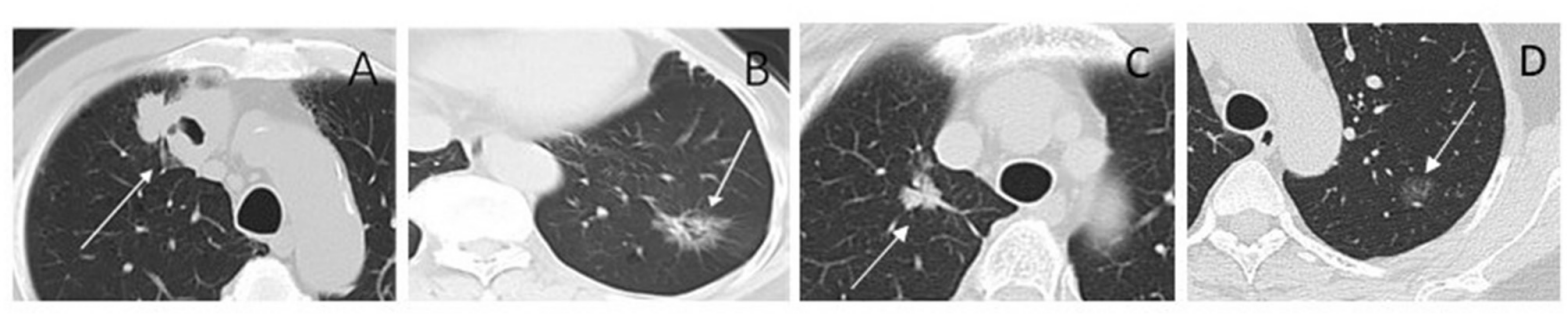
CT images of lesions for radiomic cluster analysis. **(A)** Cluster 1. Solid nodule in the right upper lobe (arrow) measuring maximum diameter 4.3 cm, predominantly solid histologic subtype, with TP53 positive, and the TMB was 13.96 mut/Mb. **(B)** Cluster 2. Part-solid nodule in the left lower lobe (arrow) measuring maximum diameter 3.0 cm, glandular vesicle-dominant histologic subtype, EGFR positive, and the TMB was 1.99 mut/Mb. **(C)** Cluster 3. Part-solid nodule in the right upper lobe (arrow) measuring maximum diameter 2.2 cm; solid and micropapillary histologic subtype; ALK-positive, and the TMB was 4.99 mut/Mb. **(D)** Cluster 4. Ground-glass nodule in the left upper lobe (arrow) measuring maximum diameter 1.3 cm; ERBB2 and PD-L1 positive; and the TMB was 5.98 mut/Mb.

Consensus clustering analysis based on radiomic features showed the most significant relative change under the conditional density function curve at a *k*-value of 4. Clinicopathology, the genetic profiles, and CT findings according to the cluster are listed in Tables 1–3. Cluster 1 (*n* = 11) mainly comprised large solid masses with a maximum diameter of 5.1 ± 2.0 cm; these cases are likely to involve tumor necrosis, thickened pleura, pleural traction, and lymph node enlargement. Almost all nodules in Cluster 1 were accompanied by lobulation. Clusters 2 and 3 were dominated by part-solid and solid masses with maximum diam eters of 2.1 ± 1.4 cm and 2.1 ± 0.9 cm, respectively; the tumors were often associated with vacuole signs, vascular cluster signs, and spiculation on CT images. Cluster 4 was mostly small ground-glass opacity lesions, with a maximum diameter of 1.0 ± 0.9 cm (*P* < 0.001). The majority of patients in Cluster 1 were in clinical stages III–IV; in Clusters 2–4, patients were mostly in early clinical stages. Differences in TMB, PD-L1 expression, and mutations in EGFR, TP53, ERBB2, BRAF, and ROS1 among the four clusters were statistically significant. Regarding targeted therapies and immunotherapies, mutations in EGFR were highest in Cluster 2 (73.1%, 95/130), followed by Cluster 3 (67.8%, 59/87). PD-L1-positive expression was highest in Cluster 1 (45.5%, 5/11), followed by Cluster 3 (28.7%, 25/87) (Figure 6). The highest TMB was in Cluster 1 (9.1 ± 4.8 mut/Mb, range: 1.99 to 14.96) (Figure 7). Representative cases are shown in Figure 8.

## 4. Discussion

In the last decade, major breakthroughs regarding the treatment of LUADs have shifted from the empirical application of cytotoxic therapy to personalized treatments based on genetic alterations and TIME. For these treatment strategies, knowledge of targeted genomics and TIME status is needed for patient selection. As both genomic sequencing and IHC require tissue specimens to be obtained through invasive processes, there is a need to find non-invasive surrogate biomarkers to facilitate the clinical translation of personalized medicine for patients with LUAD. In this study, we established imaging phenotypes of LUADs through CT-based radiomic consensus clustering with a comparison of clinicopathological metrics and targeted genomic data to guide patient selection. LUADs were clustered into four clusters according to CT-based radiomic features. When all patients were analyzed, Cluster 1 mainly consisted of large solid masses associated with advanced clinical staging (III–IV, 63.6%) with a high frequency of PD-L1 expression and TP53 mutation. CT features were more likely to be accompanied by tumor necrosis, thickened pleura, pleural traction, and lymph node enlargement. Demography shows that patients in Cluster 1 were mainly men who smoke. Clusters 2 and 3 were dominated by part-solid and solid masses associated with the EGFR mutation. These cases were likely to be associated with the vacuole sign, vascular cluster sign, and spiculation on CT images. Cluster 4 mostly consisted of GGOs. Therefore, patients in Cluster 1 might be more responsive to Immune checkpoint inhibitor (ICI) treatment, whereas tyrosine kinase inhibitors may be recommended for patients in Clusters 2 and 3. Although TMB did not correlate with PD-L1 expression in NSCLC, its elevation showed the likelihood of benefit from immunotherapy (19). Therefore, TMB has the potential to serve as a biomarker to predict response to ICI therapy in NSCLC (19, 24–26). A recent clinical trial identified that a TMB of at least 10 mut/Mb was an effective cutoff for predicting the efficacy of immunotherapy, irrespective of the tumor PD-L1 expression level (27). In this study, we observed that the difference in TMB among CT-based radiomic phenotypes was statistically significant. The highest TMB was in Cluster 1 (9.1 ± 4.8 mut/Mb, range: 1.99 to 14.96). This further supports the conclusion that Cluster 1 patients might be more suitable for immunotherapy.

Taking advantage of computer science and artificial intelligence, radiomics has made great progress in oncology to improve diagnosis, stage, prognosis, and treatment response prediction (10, 28, 29). Previous studies have shown that CT-based radiomics can predict EGFR mutation, microenvironment, and treatment response of targeted and immunotherapies in LUAD (13, 30–32). These studies highlight special molecular characteristics to develop an algorithm for a single surrogate biomarker. In clinical scenarios, comprehensive molecular characteristics are required for personalized treatment decision-making. Integration of multiple molecular characteristics might improve the predictive capacity of treatment response. Recently, Perez-Johnston et al. implemented unsupervised learning to build an image phenotype of LUADs based on CT radiomics and showed an association between imaging phenotype and genomics (15). Similarly, we identified four phenotypes using CT-based radiomic features of LUADs that correlated with genomic profiles and PD-L1 expression. In the study of Perez-Johnston et al., EGFR and STK11 mutations were statistically significant among clusters. EGFR mutation was highest in a cluster consisting of mainly sub-solid masses with solid components <10%. We also identified that the difference in EGFR mutation prevalence was statistically significant among our clusters. Cluster 2 had the highest number of EGFR mutations (73.1%), followed by Cluster 3 (67.8%). We also noted that the TP53 mutation was highest in Cluster 1, which was mainly comprised of solid masses. These findings were consistent with a previous study that reported that the TP53 mutation increased with the growth of the solid component (33). Assoun et al. found that TP53 mutations reflected TMB and were associated with immunotherapy benefits in advanced NSCLC (34). Furthermore, we found that PD-L1 expression was significantly different among clusters, with the highest expression in Cluster 1. Zu et al. analyzed TIME-related indicators by conducting a series of TIME studies. They identified emerging key biomarkers of TIME, providing new biomarkers to guide precision therapy (35–38). Therefore, the results of this study may provide guidance for targeted therapies and immunotherapies that integrate genomic profiles and the TIME of LUADs. To the best of our knowledge, this is the first comprehensive study to date to explore the association of radiomic phenotypes with the genomic profile and immune microenvironment of LUADs. Theoretically, targeted therapy might induce rapid tumor death, leading to the release of neoantigens, which in turn affect immune pathways and improve the efficacy of immunotherapy (39, 40). Thus, our study provides the possibility of immuno-targeted combination therapy, which has strong scientific merit. In addition, our study can provide generalizable guidance across various treatment settings.

Simultaneously, we also observed that mutations in BRAF, ROS1, MET, and ERBB2 were statistically significant. Although there are currently no targeted therapies for tumors with these mutations, multiple clinical trials are underway to evaluate the efficacy of targeting these genes in cancers, and this finding might provide guidance for future targeted medicine research.

There were several limitations in this study. First, this was a retrospective study performed at a single institution, and the small sample size might limit the generalizability of the findings. Cluster 1 had a small sample size of only 11 cases. Therefore, a multicenter study including more patients and prospective validation is warranted to improve the model's robustness. Second, the reproducibility of our findings and their clinical implications may be challenging in a more diverse clinical context, as our study only included clinical lung cancer patients who underwent tumor resection. The clinical utility of radiomics needs to be further established through rigorous clinical validation studies. Third, only a few patients received targeted therapy or immunotherapy. The association between the imaging phenotypes and treatment response was not probed. Finally, ROI drawing was manual rather than semi-automatic or automatic, which might be operator-dependent.

In conclusion, CT-based radiomic phenotypes were able to identify LUADs with different molecular characteristics non-invasively, showing the potential to provide treatment decision-making support for clinicians about patients with LUAD. Radiogenomics is still at an early stage of research, and future efforts are needed to optimize its methods and standardize its processes. In future clinical environments, integrating radiogenomics into existing workflows may add value to conventional imaging to facilitate personalized medicine in patients with LUAD.

## Data availability statement

The original contributions presented in the study are included in the article/supplementary material, further inquiries can be directed to the corresponding author.

## Ethics statement

The studies involving human participants were reviewed and approved by Institutional Review Board (Shenzhen People's Hospital). Written informed consent for participation was not required for this study in accordance with the national legislation and the institutional requirements.

## Author contributions

XL completed the study design, data collection, statistical analysis, article writing, and chart making. TX, SW, and YC extracted the radiomic features from CT images and analyzed the data. TX and WX participated in the data collection. JG and CJ participated in the revision of the manuscript. All authors read and approved the final manuscript and contributed to the article and approved the submitted version.

## References

[1] Sung H Ferlay J Siegel RL Laversanne M Soerjomataram I Jemal A. Global cancer statistics 2020: GLOBOCAN estimates of incidence and mortality worldwide for 36 cancers in 185 countries. CA Cancer J Clin. (2021) 71:209–49. 10.3322/caac.21660 33538338

[2] Siegel RL Miller KD Fuchs HE Jemal A. Cancer statistics, 2021. CA Cancer J Clin. (2021) 71:7–33. 10.3322/caac.2165 33433946

[3] Planchard D Popat S Kerr K Novello S Smit EF Faivre-Finn C. Metastatic non-small cell lung cancer: ESMO clinical practice guidelines for diagnosis, treatment and follow-up. Ann Oncol. (2018) 29:iv192–237. 10.1093/annonc/mdy275 30285222

[4] Santabarbara G Maione P Rossi A Palazzolo G Gridelli C. The role of pembrolizumab in the treatment of advanced non-small cell lung cancer. Ann Transl Med. (2016) 4:215. 10.21037/atm.2016.05.64 27386489 PMC4916358

[5] Miller M Hanna N. Advances in systemic therapy for non-small cell lung cancer. BMJ. (2021) 375:n2363. 10.1136/bmj.n2363 34753715

[6] Ettinger DS Wood DE Aggarwal C Aisner DL Akerley W Bauman JR. NCCN guidelines insights: non-small cell lung cancer, version 1.2020. J Natl Compr Canc Netw. (2019) 17:1464–72. 10.6004/jnccn.2019.0059 31805526

[7] Genova C Dellepiane C Carrega P Sommariva S Ferlazzo G Pronzato P. Therapeutic implications of tumor microenvironment in lung cancer: focus on immune checkpoint blockade. Front Immunol. (2021) 12:799455. 10.3389/fimmu.2021.799455 35069581 PMC8777268

[8] Herbst RS Baas P Kim DW Felip E Perez-Gracia JL Han JY. Pembrolizumab versus docetaxel for previously treated, PD-L1-positive, advanced non-small-cell lung cancer (KEYNOTE-010): a randomised controlled trial. Lancet. (2016) 387:1540–50. 10.1016/S0140-6736(15)01281-7 26712084

[9] Huang WL Chen YL Yang SC Ho CL Wei F Wong DT. Liquid biopsy genotyping in lung cancer: ready for clinical utility? Oncotarget. (2017) 8:18590–608. 10.18632/oncotarget.14613 28099915 PMC5392351

[10] Gillies RJ Kinahan PE Hricak H. Radiomics: images are more than pictures, they are data. Radiology. (2016) 278:563–77. 10.1148/radiol.2015151169 26579733 PMC4734157

[11] Lambin P Leijenaar RTH Deist TM Peerlings J de Jong EEC van Timmeren J. Radiomics: the bridge between medical imaging and personalized medicine. Nat Rev Clin Oncol. (2017) 14:749–62. 10.1038/nrclinonc.2017.141 28975929

[12] Yang L He YT Dong S Wei XW Chen ZH Zhang B. Single-cell transcriptome analysis revealed a suppressive tumor immune microenvironment in EGFR mutant lung adenocarcinoma. J Immunother Cancer. (2022) 10:3534. 10.1136/jitc-2021-003534 35140113 PMC8830346

[13] Xie D Wang TT Huang SJ Deng JJ Ren YJ Yang Y. Radiomics nomogram for prediction disease-free survival and adjuvant chemotherapy benefits in patients with resected stage I lung adenocarcinoma. Transl Lung Cancer Res. (2020) 9:1112–23. 10.21037/tlcr-19-577 32953490 PMC7481634

[14] Jiang C Luo Y Yuan J You S Chen Z Wu M. CT-based radiomics and machine learning to predict spread through air space in lung adenocarcinoma. Eur Radiol. (2020) 30:4050–7. 10.1007/s00330-020-06694-z 32112116

[15] Perez-Johnston R Araujo-Filho JA Connolly JG Caso R Whiting K Tan KS. CT-based radiogenomic analysis of clinical stage I lung adenocarcinoma with histopathologic features and oncologic outcomes. Radiology. (2022) 303:664–72. 10.1148/radiol.211582 35230187 PMC9131171

[16] Doroshow DB Bhalla S Beasley MB Sholl LM Kerr KM Gnjatic S. PD-L1 as a biomarker of response to immune-checkpoint inhibitors. Nat Rev Clin Oncol. (2021) 18:345–62. 10.1038/s41571-021-00473-5 33580222

[17] Zhou J Sanchez-Vega F Caso R Tan KS Brandt WS Jones GD. Analysis of tumor genomic pathway alterations using broad-panel next-generation sequencing in surgically resected lung adenocarcinoma. Clin Cancer Res. (2019) 25:7475–84. 10.1158/1078-0432.CCR-19-1651 31455678 PMC6911636

[18] Cheng DT Mitchell TN Zehir A Shah RH Benayed R Syed A. Memorial sloan kettering-integrated mutation profiling of actionable cancer targets (MSK-IMPACT): a hybridization capture-based next-generation sequencing clinical assay for solid tumor molecular oncology. J Mol Diagn. (2015) 17:251–64. 10.1016/j.jmoldx.2014.12.006 25801821 PMC5808190

[19] Rizvi H Sanchez-Vega F La K Chatila W Jonsson P Halpenny D. Molecular determinants of response to anti-programmed cell death (PD)-1 and anti-programmed death-ligand 1 (PD-L1) blockade in patients with non-small-cell lung cancer profiled with targeted next-generation sequencing. J Clin Oncol. (2018) 36:633–41. 10.1200/JCO.2017.75.3384 29337640 PMC6075848

[20] Shen R Seshan VE FACETS. allele-specific copy number and clonal heterogeneity analysis tool for high-throughput DNA sequencing. Nucleic Acids Res. (2016) 44:e131. 10.1093/nar/gkw520 27270079 PMC5027494

[21] Aoki T Nakata H Watanabe H Nakamura K Kasai T Hashimoto H. Evolution of peripheral lung adenocarcinomas: CT findings correlated with histology and tumor doubling time. AJR Am J Roentgenol. (2000) 174:763–8. 10.2214/ajr.174.3.1740763 10701622

[22] Hansell DM Bankier AA MacMahon H McLoud TC Muller NL Remy J. Fleischner Society: glossary of terms for thoracic imaging. Radiology. (2008) 246:697–722. 10.1148/radiol.2462070712 18195376

[23] Hsu JS Huang MS Chen CY Liu GC Liu TC Chong IW. Correlation between EGFR mutation status and computed tomography features in patients with advanced pulmonary adenocarcinoma. J Thorac Imaging. (2014) 29:357–63. 10.1097/RTI.0000000000000116 25303964

[24] Samstein RM Lee CH Shoushtari AN Hellmann MD Shen R Janjigian YY. Tumor mutational load predicts survival after immunotherapy across multiple cancer types. Nat Genet. (2019) 51:202–6. 10.1038/s41588-018-0312-8 30643254 PMC6365097

[25] Chan TA Yarchoan M Jaffee E Swanton C Quezada SA Stenzinger A. Development of tumor mutation burden as an immunotherapy biomarker: utility for the oncology clinic. Ann Oncol. (2019) 30:44–56. 10.1093/annonc/mdy495 30395155 PMC6336005

[26] High TMB. Predicts immunotherapy benefit. Cancer Discov. (2018) 8:668. 10.1158/2159-8290.CD-NB2018-048 29661758

[27] Hellmann MD Ciuleanu TE Pluzanski A Lee JS Otterson GA Audigier-Valette C. Nivolumab plus ipilimumab in lung cancer with a high tumor mutational burden. N Engl J Med. (2018) 378:2093–104. 10.1056/NEJMoa1801946 29658845 PMC7193684

[28] Shi L He Y Yuan Z Benedict S Valicenti R Qiu J. Radiomics for response and outcome assessment for non-small cell lung cancer. Technol Cancer Res Treat. (2018) 17:1533033818782788. 10.1177/1533033818782788 29940810 PMC6048673

[29] Huang YQ Liang CH He L Tian J Liang CS Chen X. Development and validation of a radiomics nomogram for preoperative prediction of lymph node metastasis in colorectal cancer. J Clin Oncol. (2016) 34:2157–64. 10.1200/JCO.2015.65.9128 27138577

[30] Zhu Y Guo YB Xu D Zhang J Liu ZG Wu X. A computed tomography (CT)-derived radiomics approach for predicting primary co-mutations involving TP53 and epidermal growth factor receptor (EGFR) in patients with advanced lung adenocarcinomas (LUAD). Ann Transl Med. (2021) 9:545. 10.21037/atm-20-6473 33987243 PMC8105857

[31] Zhang X Lu B Yang X Lan D Lin S Zhou Z. Prognostic analysis and risk stratification of lung adenocarcinoma undergoing EGFR-TKI therapy with time-serial CT-based radiomics signature. Eur Radiol. (2023) 33:825–35. 10.1007/s00330-022-09123-5 36166088 PMC9889474

[32] Lee G Park H Sohn I Lee SH Song SH Kim H. Comprehensive Computed Tomography Radiomics Analysis Of Lung Adenocarcinoma For Prognostication. Oncologist. (2018) 23:806–13. 10.1634/theoncologist.2017-0538 29622699 PMC6058328

[33] Aoki T Hanamiya M Uramoto H Hisaoka M Yamashita Y Korogi Y. Adenocarcinomas with predominant ground-glass opacity: correlation of morphology and molecular biomarkers. Radiology. (2012) 264:590–6. 10.1148/radiol.12111337 22653188

[34] Assoun S Theou-Anton N Nguenang M Cazes A Danel C Abbar B. Association of TP53 mutations with response and longer survival under immune checkpoint inhibitors in advanced non-small-cell lung cancer. Lung Cancer. (2019) 132:65–71. 10.1016/j.lungcan.2019.04.005 31097096

[35] Cai Z Chen J Yu Z Li H Liu Z Deng D. BCAT2 shapes a non-inflamed tumor microenvironment and induces resistance to anti-PD-1/PD-L1 immunotherapy by negatively regulating proinflammatory chemokines and anticancer immunity. Adv Sci (Weinh). (2023) 10:e2207155. 10.1002/advs.202207155 36642843 PMC10015882

[36] Hu J Othmane B Yu A Li H Cai Z Chen X. 5mC regulator-mediated molecular subtypes depict the hallmarks of the tumor microenvironment and guide precision medicine in bladder cancer. BMC Med. (2021) 19:289. 10.1186/s12916-021-02163-6 34836536 PMC8627095

[37] Hu J Yu A Othmane B Qiu D Li H Li C. Siglec15 shapes a non-inflamed tumor microenvironment and predicts the molecular subtype in bladder cancer. Theranostics. (2021) 11:3089–108. 10.7150/thno.53649 33537076 PMC7847675

[38] Hu J Chen J Ou Z Chen H Liu Z Chen M. Neoadjuvant immunotherapy, chemotherapy, and combination therapy in muscle-invasive bladder cancer: a multi-center real-world retrospective study. Cell Rep Med. (2022) 3:100785. 10.1016/j.xcrm.2022.100785 36265483 PMC9729796

[39] Vanneman M Dranoff G. Combining immunotherapy and targeted therapies in cancer treatment. Nat Rev Cancer. (2012) 12:237–51. 10.1038/nrc3237 22437869 PMC3967236

[40] Sharma P Allison JP. Immune checkpoint targeting in cancer therapy: toward combination strategies with curative potential. Cell. (2015) 161:205–14. 10.1016/j.cell.2015.03.030 25860605 PMC5905674

